# Efficacy and Safety of Rb‐bFGF in Hair Transplantation: A Prospective and Comparative Study

**DOI:** 10.1111/jocd.70464

**Published:** 2025-09-18

**Authors:** Jie Lei, Xianghao Xu, Yanping Wang, Zhonglian An, Zhen Liang, Ping Xue

**Affiliations:** ^1^ Department of Plastic Surgery Xijing Hospital, Fourth Military Medical University Xi'an China; ^2^ Department of Medicine Xi'an PLA Hospital Xi'an China; ^3^ Emergency Department Jiangjin Hospital Affiliated to Chongqing University Chongqing China

**Keywords:** clinical application, combined treatment, fibroblast growth factor, hair transplantation, prospective study

## Abstract

**Objective:**

This prospective randomized controlled trial aimed to evaluate the clinical value of recombinant bovine basic fibroblast growth factor (rb‐bFGF)‐based combined therapy in hair transplantation, focusing on its optimization effects on follicle survival rate, postoperative hair‐loss dynamics, complication control, and patient satisfaction.

**Methods:**

Sixty patients with moderate‐to‐severe androgenetic alopecia were randomly assigned to either an rb‐bFGF group or a control group. In the rb‐bFGF group, harvested follicles were intraoperatively immersed in an rb‐bFGF–enriched solution and patients received postoperative topical rb‐bFGF gel for 3 weeks, in addition to baseline medications and minoxidil. Control group follicles were stored in saline, with postoperative minoxidil alone. Quantitative assessments include measurements of hair follicle viability and hair density at 3, 6, and 12 months postoperatively. Patient satisfaction was assessed using a three‐level scale, and complication rates were recorded.

**Results:**

The rb‐bFGF group demonstrated superior outcomes compared with controls, including higher 12‐month follicle survival (91.1% vs. 81.0%), lower hair‐loss rate (11.6% vs. 22.7%), greater patient satisfaction (96.7% vs. 80.0%), and fewer complications (20.0% vs. 85.3%). All differences were statistically significant (*p* < 0.001).

**Conclusion:**

Intraoperative follicle immersion and postoperative topical application of rb‐bFGF significantly enhance graft survival, reduce early hair loss, decrease complications, and improve overall outcomes in hair transplantation.

## 1 Introduction

Hair loss has become a significant global public health issue, with the Global Burden of Disease study reporting over 2.3 billion people affected, a number that continues to rise [1, 2]. While not typically life‐threatening, hair loss has profound psychosocial impacts, increasing the risk of depression by 2.3 times and affecting mental health [3]. It can lead to emotional distress, such as low self‐esteem and anxiety, and impair daily social interactions and quality of life [4]. Socially, it may hinder career advancement, personal relationships, and social integration, posing a broader challenge to societal stability and harmony [5, 6].

Clinically, interventions for hair loss face several challenges [7]. For instance, minoxidil, a common topical treatment, requires at least 6 months of consistent use to show effectiveness and has a relatively high relapse rate within 12 months posttreatment, burdening patients with long‐term use [8, 9]. Follicular unit extraction (FUE), while offering a permanent hair‐loss solution, also has limitations [10]. In scarred areas, microcirculation issues can reduce graft survival rates, compromizing overall hair‐transplantation outcomes and patient satisfaction [11, 12].

Recent advances in biotechnology have made growth factor therapy a promising new direction [13, 14]. The fibroblast growth factor (FGF) family, in particular, plays a key role in hair follicle biology [15]. By activating the FGFR1 receptor and modulating the ERK/PI3K‐AKT signaling pathway, FGF directly promotes the proliferation of hair follicle stem cells and angiogenesis [16]. This creates a favorable microenvironment for follicle growth and repair [17, 18]. In contrast to autologous biologics such as platelet‐rich plasma (PRP) and plasma rich in growth factors (PRGF), which contain heterogeneous mixtures of cytokines and show marked interindividual variability, recombinant bovine basic fibroblast growth factor (rb‐bFGF) provides a standardized, high‐purity agent with a defined mechanism of action. It directly activates FGFR1 to stimulate angiogenesis and follicle regeneration, offering greater predictability, dosing precision, and stability than PRP or PRGF. Moreover, its established safety profile in wound repair and ocular surface healing underscores its translational relevance [16]. However, current clinical applications of growth factor therapy have limitations, such as low bioavailability of topical formulations due to the epidermal barrier, which restricts drug concentration and duration at the follicle site [19, 20].

Against this backdrop, our prospective randomized controlled trial pioneers in evaluating the clinical value of recombinant bovine basic fibroblast growth factor (rb‐bFGF) in hair‐transplantation patients. A multidimensional assessment system was designed to comprehensively evaluate treatment outcomes, offering high‐quality evidence for precision treatment in this field. This study not only promotes a shift in hair‐transplantation technology toward “functional regeneration” but also provides valuable insights for future research and clinical practice, bringing new hope to hair‐loss patients.

## 2 Methods

### 2.1 Research Subjects and Treatment Methods

This single‐center prospective randomized controlled trial ran from January 2023 to June 2024 and included 60 patients with moderate‐to‐severe androgenetic alopecia (AGA). Randomization was performed using a computer‐generated random sequence without stratification by sex or alopecia stage. Allocation concealment was ensured with sequentially numbered, opaque, sealed envelopes. These envelopes were prepared and maintained by an independent research coordinator who was not involved in patient recruitment, surgery, or outcome assessment, and were opened only after patient enrollment to assign participants to groups. Each group had 30 patients, and baseline characteristics showed no significant differences (*p* > 0.05) (Table 1). This research was approved by the hospital ethics committee, and all patients gave written informed consent. The study followed the Declaration of Helsinki and relevant medical data management regulations to protect patient rights and ensure legal and compliant data handling.

**TABLE 1:** Comparison of baseline characteristics between the two groups of patients.

Variable	Study group (*n* = 30)	Control group (*n* = 30)	*t*/*χ* ^2^	*p*
Age (years, mean ± SD)	49.25 ± 9.15	48.92 ± 8.73	0.143	0.887
Male (*n*, %)	17 (56.67%)	16 (53.33%)	0.067	0.795
Norwood–Hamilton	4.15 ± 1.05	4.07 ± 0.98	0.306	0.761
Ludwig	1.87 ± 0.95	1.93 ± 0.92	0.249	0.805
Cardiovascular disease	6 (20.00%)	6 (20.00%)	0.000	1.000
Diabetes	1 (3.33%)	3 (10.00%)	1.071	0.301
Smoking	3 (10.00%)	4 (13.33%)	0.162	0.687
BMI (kg/m^2^)	28.68 ± 5.11	28.53 ± 4.95	0.116	0.909
PLT × 10^9^/L	207.85 ± 78.92	203.27 ± 75.45	0.230	0.819

Inclusion criteria were: (1) aged 25–55; (2) diagnosed with progressive AGA for ≥ 2 years; (3) occipital donor‐area hair density ≥ 80 FU/cm^2^; and (4) agreement to 12‐month follow‐up. Exclusion criteria were: (1) keloid or active scalp infection; (2) hair treatment in the past 6 months; (3) coagulation disorders; (4) pregnant or breastfeeding women; and (5) systemic diseases like thyroid dysfunction. The termination criterion was severe postoperative adverse reactions or ≥ 3 missed follow‐ups.

### 2.2 Intervention Protocol

In terms of basic medication, male patients received oral finasteride (1 mg/d) starting on the day of surgery, while female patients received oral spironolactone (20 mg, twice daily). The planned treatment duration was 2 years. Male patients could reduce the dose to 0.5 mg/day for lifelong maintenance after 2 years, and female patients underwent gradual discontinuation. Patients in both groups initiated topical minoxidil once daily during the first postoperative week, increasing to twice daily thereafter.

For preoperative preparation, all patients underwent standardized donor‐area hair preprocessing 48 h before surgery, retaining 1 mm of extradermal hair shaft length. High‐resolution imaging (EOS 6D Mark II, Canon, Japan) was used for three‐dimensional mapping of alopecia areas. A transparent grid film established a coordinate system with 1 cm^2^ unit grids to precisely quantify the baldness area. Extraction boundaries in the occipital safe donor area were calculated based on a preset follicular unit (FU) demand model (30–35 FU/cm^2^).

Regarding intraoperative procedures, surgery was performed under local tumescent anesthesia using a solution containing 1:100 000 epinephrine (5 g/L lidocaine + 1.875 g/L bupivacaine). A surgeon with ≥ 3 years of hair transplantation experience operated the Zhongmei Zhiguang system with a 0.80 mm sharp punch. Key parameters were rigorously controlled: Insertion angle maintained at (15 ± 2)° parallel to the follicular axis, extraction depth monitored in real time via ultrasound and confined to 5–8 mm, and donor density ≤ 15 FU/cm^2^. Extracted follicles underwent dual‐microscopy quality control to ensure a transection rate < 5%. Follicular processing was group‐specific: control group follicles were stored in saline‐soaked gauze at 4°C; study group follicles were immersed in a specialized low‐temperature (4°C) nutrient suspension (saline + dissolved rb‐bFGF [21 000 IU/5 g, Zhuhai Yisheng]) until implantation. Postoperatively, the rb‐bFGF group additionally received topical rb‐bFGF gel (21 000 IU/5 g) applied four times daily to the recipient area from postoperative day 1 through day 21, with minoxidil usage identical to that of the control group. All patients maintained their baseline oral medication (finasteride for males/spironolactone for females) and synchronized minoxidil topical therapy.

Recipient site implantation used 22G micro‐implants (LeadM, Korea), with angles designed according to scalp biomechanics: frontal zone at 30°–45° to mimic natural hairlines, vertex zone at 40°–60° to align with whorl patterns. Density was dynamically calibrated to (35 ± 2) FU/cm^2^ via computer navigation (Navilock FUE 3.0) to ensure spatial homogeneity.

For postoperative management, both groups had dressings removed and wounds gently cleansed (i.e., local exposure) 24 h postoperatively. The control group used 5% minoxidil tincture (Zhendong) once daily during the first postoperative week, then twice daily, maintaining treatment through day 180.

### 2.3 Evaluation Criteria and Quality Control

A multidimensional efficacy‐evaluation system was established, covering objective measurements and subjective assessments. At 3, 6, and 12 months postoperatively, the Folliscope 5.0 high‐resolution imaging system (LeadM, Korea) quantitatively assessed hair‐loss progression. In three fixed 1 cm^2^ mesh areas in the transplant zone, the hair‐loss rate was calculated as: (immediate postoperative density − follow‐up density)/immediate postoperative density × 100%. Long‐term follicle survival was verified during follow‐ups using the complementary survival rate formula: survival rate = follow‐up density/immediate postoperative density × 100%. These two metrics provided core objective evidence of follicle stability. In addition, a positive hair‐strength reaction was defined as abnormal stiffness and resistance detected when gently pulling transplanted hairs during the early postoperative months. This finding suggests premature follicle‐cycle regression and was used as a surrogate marker of compromised graft quality.

Patient‐reported experience was collected via a three‐grade satisfaction scale (very satisfied/satisfied/dissatisfied), recorded by an independent physician through structured interviews. The satisfaction rate was calculated as: (number of very satisfied + satisfied)/total cases × 100%. Safety monitoring focused on six specific complications (hematoma, folliculitis, surgical‐site pain, pruritus, curly hair, and skin necrosis), recording the first occurrence within 12 months. The total complication rate was the proportion of patients with ≥ 1 adverse event. All measurements followed a standardized manual and were performed by a blinded assessment team.

A triple‐blind design was implemented: (1) statisticians generated random sequences independently; (2) assessors were unaware of treatment allocation; and (3) data entry was verified by dual‐data entry. Transplanted‐follicle quality was confirmed by dual‐microscopic review (transverse‐section rate ≤ 3% was acceptable), and postoperative follow‐up consistency was assessed by two attending physicians (Kappa value = 0.92).

### 2.4 Statistical Analysis

Data were analyzed using SPSS 26.0. Measurement data are presented as mean ± SD, with intergroup comparisons by independent‐samples *t*‐test. Categorical data are presented as frequency (%), analyzed by *χ* ^2^ test or Fisher's exact test. *p* < 0.05 was considered statistically significant.

## 3 Results

### 3.1 Comparison of Baseline Characteristics

The baseline characteristics of the two groups of patients, including age, male, Norwood–Hamilton, Ludwig, cardiovascular disease, diabetes, smoking, BMI, and PLT, were compared. The results indicated that there were no significant differences in baseline characteristics between the two groups (*p* > 0.05), as shown in Table 1.

### 3.2 Comparison of Transplantation Outcomes

Postoperative evaluations revealed that the study group significantly outperformed the control group in key hair transplantation metrics. Although the number of transplanted follicular units was comparable between the two groups, the study group achieved a higher transplantation density (33.81 ± 1.95 per cm^2^) and longer growth duration (20.33 ± 2.81 days). Notably, the study group exhibited a significantly lower rate of positive hair‐strength reactions (10.00% vs. 46.67%, *p* < 0.001), as shown in Table 2.

**TABLE 2:** Comparison of transplantation outcomes between the two groups of patients.

Variable	Study group (*n* = 30)	Control group (*n* = 30)	*t*/*χ* ^2^	*p*
Number of transplanted follicular units	1001.42 ± 304.83	1005.73 ± 298.67	0.055	0.956
Transplantation density (units/cm^2^)	33.81 ± 1.95	31.59 ± 1.83	4.547	< 0.001
Growth duration (days)	20.33 ± 2.81	17.38 ± 2.19	4.535	< 0.001
Positive hair‐strength reactions (*n*, %)	3 (10.00%)	14 (46.67%)	10.345	< 0.001

### 3.3 Dynamic Efficacy Monitoring

Postoperative dynamic monitoring showed that the study group exhibited superior efficacy stability throughout the hair transplantation process. At 3 months postoperatively, the study group had a hair‐loss rate of 33.92%, approximately half that of the control group. This advantage persisted and expanded over time, with the study group's hair‐loss rate stabilizing at 11.63% by 12 months, significantly lower than the control group (*p* < 0.001). Regarding follicle survival, the study group achieved a 3‐month survival rate of 96.08%. While both groups experienced declines in survival rates over time, the study group maintained a high survival rate above 90%, significantly higher than the control group (*p* < 0.001), as shown in Table 3.

**TABLE 3:** Comparison of hair‐loss and follicle survival dynamics between the two groups.

Indicator	Time point	Study group (*n* = 30)	Control group (*n* = 30)	*t*	*p*
Hair‐loss rate	3 months	33.92 ± 1.85	67.53 ± 2.79	54.991	< 0.001
	6 months	14.67 ± 2.15	34.22 ± 2.41	33.155	< 0.001
	12 months	11.63 ± 2.18	22.71 ± 2.17	19.729	< 0.001
Follicle survival rate	3 months	96.08 ± 2.85	89.12 ± 5.08	6.545	< 0.001
	6 months	93.17 ± 3.55	85.14 ± 5.03	7.144	< 0.001
	12 months	91.12 ± 2.81	80.98 ± 6.88	7.473	< 0.001

### 3.4 Analysis of Patient Treatment Satisfaction

Patient‐reported outcome measures indicated that the study group had significantly higher treatment satisfaction. The overall satisfaction rate in the study group was 96.67%, markedly higher than the 80.00% in the control group (*p* < 0.001). This suggests that combined therapy not only improves objective efficacy but also enhances patients' subjective treatment experience and acceptance, as shown in Table 4.

**TABLE 4:** Comparison of postoperative satisfaction ratings between the two groups.

Satisfaction level	Study group (*n* = 30)	Control group (*n* = 30)	*χ* ^2^	*p*
Very satisfied	22 (73.33%)	10 (33.33%)		
Satisfied	7 (23.33%)	14 (46.67%)		
Dissatisfied	1 (3.33%)	6 (20.00%)		
Satisfaction rate	29 (96.67%)	24 (80.00%)	4.043	< 0.001

### 3.5 Evaluation of Treatment Safety

Postoperative safety assessments revealed that the study group had a significant advantage in complication control, with a total complication rate of 20.00% in the study group compared to 83.33% in the control group (*p* < 0.001), as shown in Table 5.

**TABLE 5:** Comparison of postoperative complication rates between the two groups of patients.

Complication type	Study group (*n* = 30)	Control group (*n* = 30)	*χ* ^2^	*p*
Hematoma	1 (3.33%)	5 (16.67%)		
Folliculitis	1 (3.33%)	4 (13.33%)		
Surgical‐site pain	2 (6.67%)	6 (20.00%)		
Pruritus	1 (3.33%)	5 (16.67%)		
Curly hair	1 (3.33%)	3 (10.00%)		
Skin necrosis	0 (0.00%)	2 (6.67%)		
Total	6 (20.00%)	25 (83.33%)	27.273	< 0.001

## 4 Cases Report

### 4.1 Case 1

**FIGURE 1:**
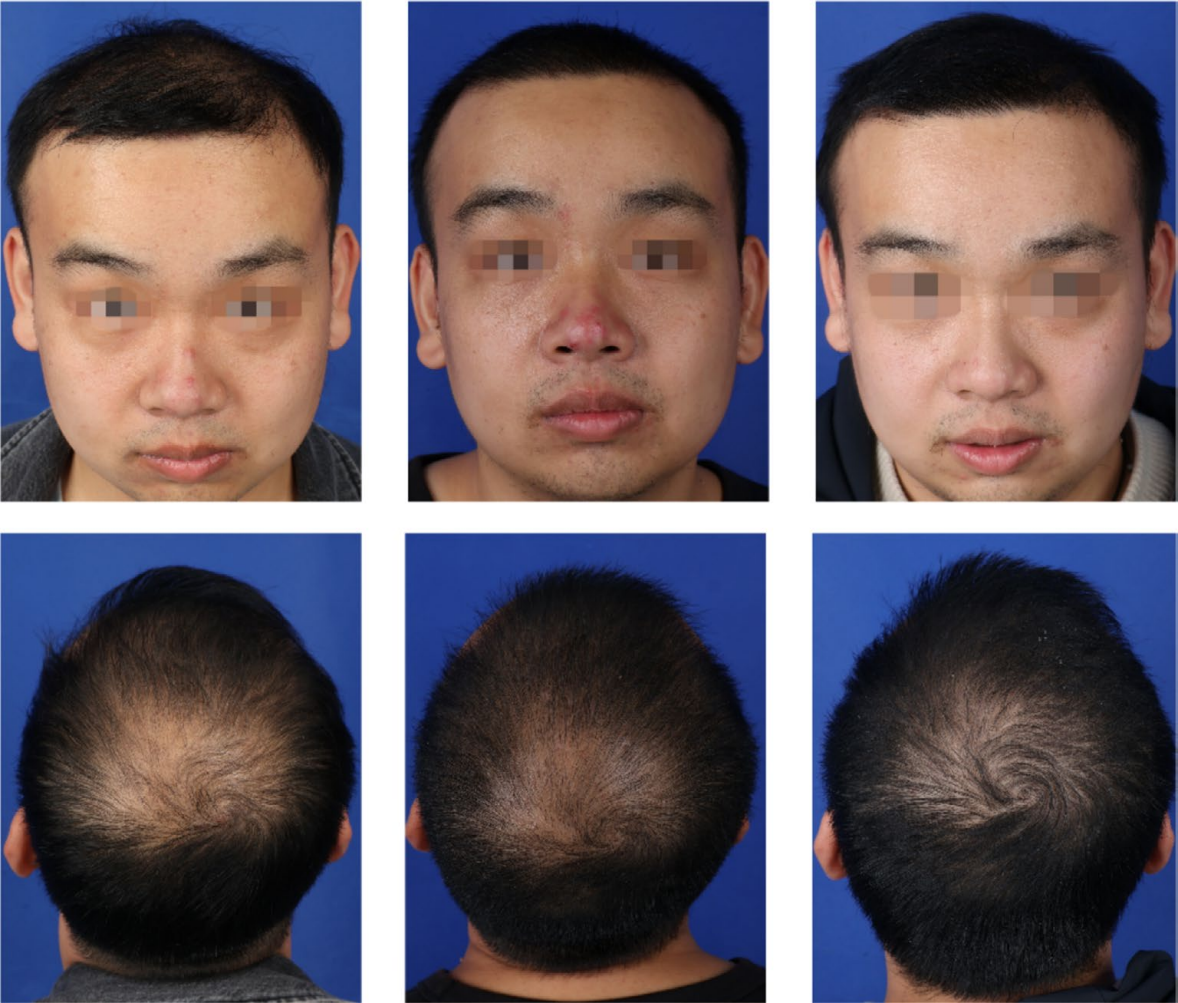
Typical case image of control group patient. Photographs of the control group patient who underwent FUE treatment alone are presented at the preoperative stage (left), and at 6 months (middle) and 12 months (right) post‐procedure. Postoperative evaluation demonstrated partial regrowth of hair from the transplanted follicles, although certain areas continued to appear sparse and less dense.

A 26‐year‐old male patient presented with a 5‐year history of progressive thinning of hair at the crown, with a hair‐loss area of 48 cm^2^. He underwent FUE hair transplantation without the use of growth factors, during which 1260 follicular units were transplanted at a density of 34.8 units per cm^2^. Postoperatively, his hair‐loss rates were 65.3% at 3 months and 32.1% at 6 months, with a 12‐month follicle survival rate of 82.4%. The patient reported experiencing intermittent pain in the occipital donor area and expressed basic satisfaction with the hair density achieved on his crown (Figure 1).

### 4.2 Case 2

**FIGURE 2:**
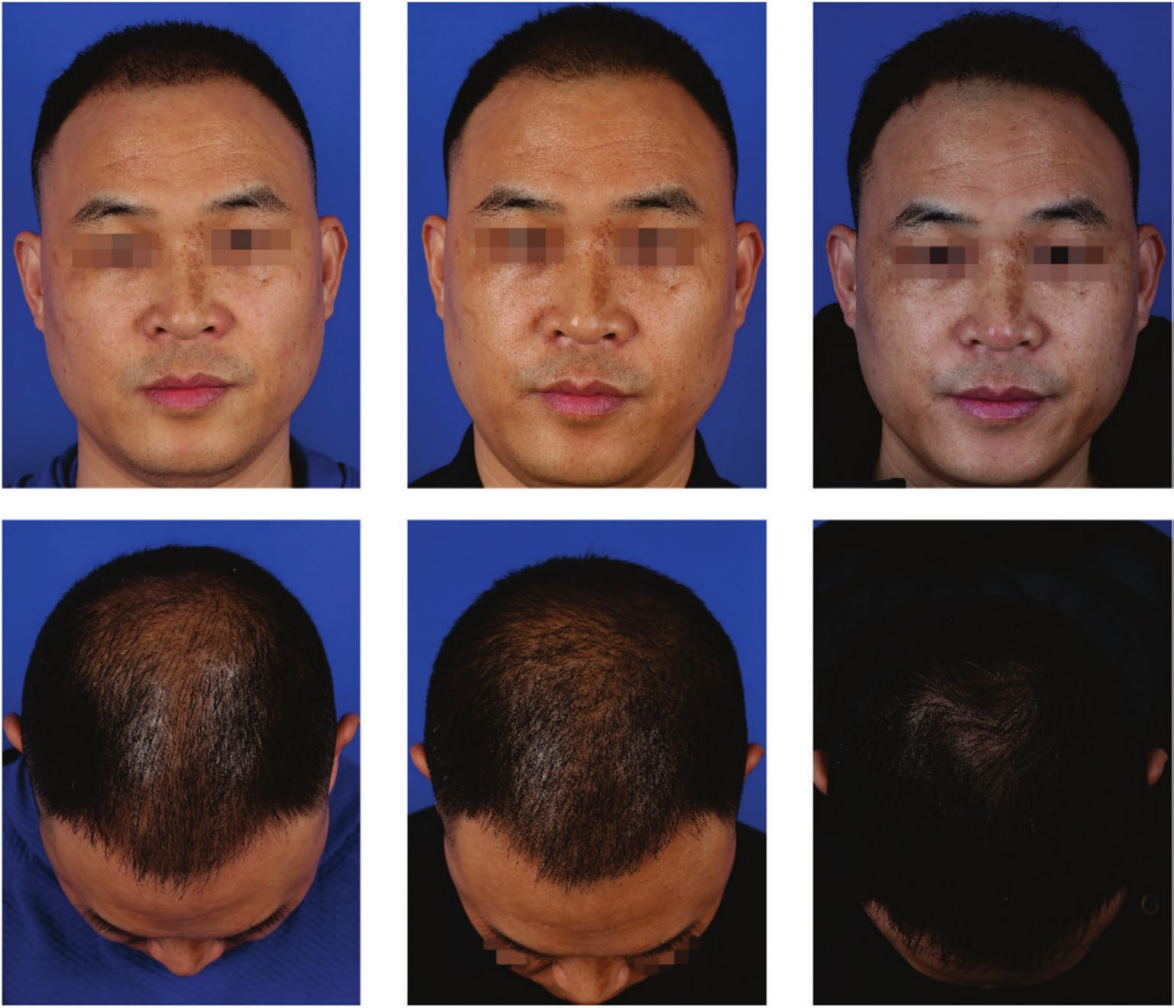
Typical case image of study group patient. Photographs of the experimental group patient who received combined rb‐bFGF and FUE treatment are shown at the preoperative stage (left), 6 (middle), and 12 (right) months posttreatment. Postoperative evaluations revealed significantly increased hair density, demonstrating excellent therapeutic outcomes.

A 41‐year‐old male patient presented with a 12‐year history of progressive hair thinning on the vertex, with a hair‐loss area measuring approximately 38 cm^2^. He underwent FUE hair transplantation combined with rb‐bFGF follicle soaking and postoperative topical application. A total of 920 follicular units were transplanted at a density of 35.2 units/cm^2^. Following surgery, his hair‐loss rates were recorded at 32.7% at 3 months, 14.8% at 6 months, and 11.2% at 12 months, with follicle survival rates steadily improving to 94.1%, 90.6%, and 89.8%, respectively. No complications such as folliculitis were observed during follow‐up. The patient reported being highly satisfied with the results of the hair restoration procedure (Figure 2).

### 4.3 Case 3

A 29‐year‐old female patient in the experimental group presented with progressive thinning of hair at the crown and mild frontal hairline recession. Her condition was classified as Ludwig grade II, with a hair loss area of approximately 46 cm^2^. Following evaluation, she was diagnosed with androgenetic alopecia (AGA). She underwent FUE hair transplantation combined with intraoperative rb‐bFGF follicle soaking and postoperative topical application. A total of 1020 follicular units were transplanted at a density of 35.2 units/cm^2^. Postoperatively, her hair‐loss rates were 32.7% at 3 months, 13.5% at 6 months, and 10.2% at 12 months, with corresponding follicle survival rates of 95.8%, 93.4%, and 91.1%, respectively. No adverse reactions, such as folliculitis, were observed during the follow‐up period. The patient reported being highly satisfied with the outcome of her hair restoration procedure.

**Figure:**
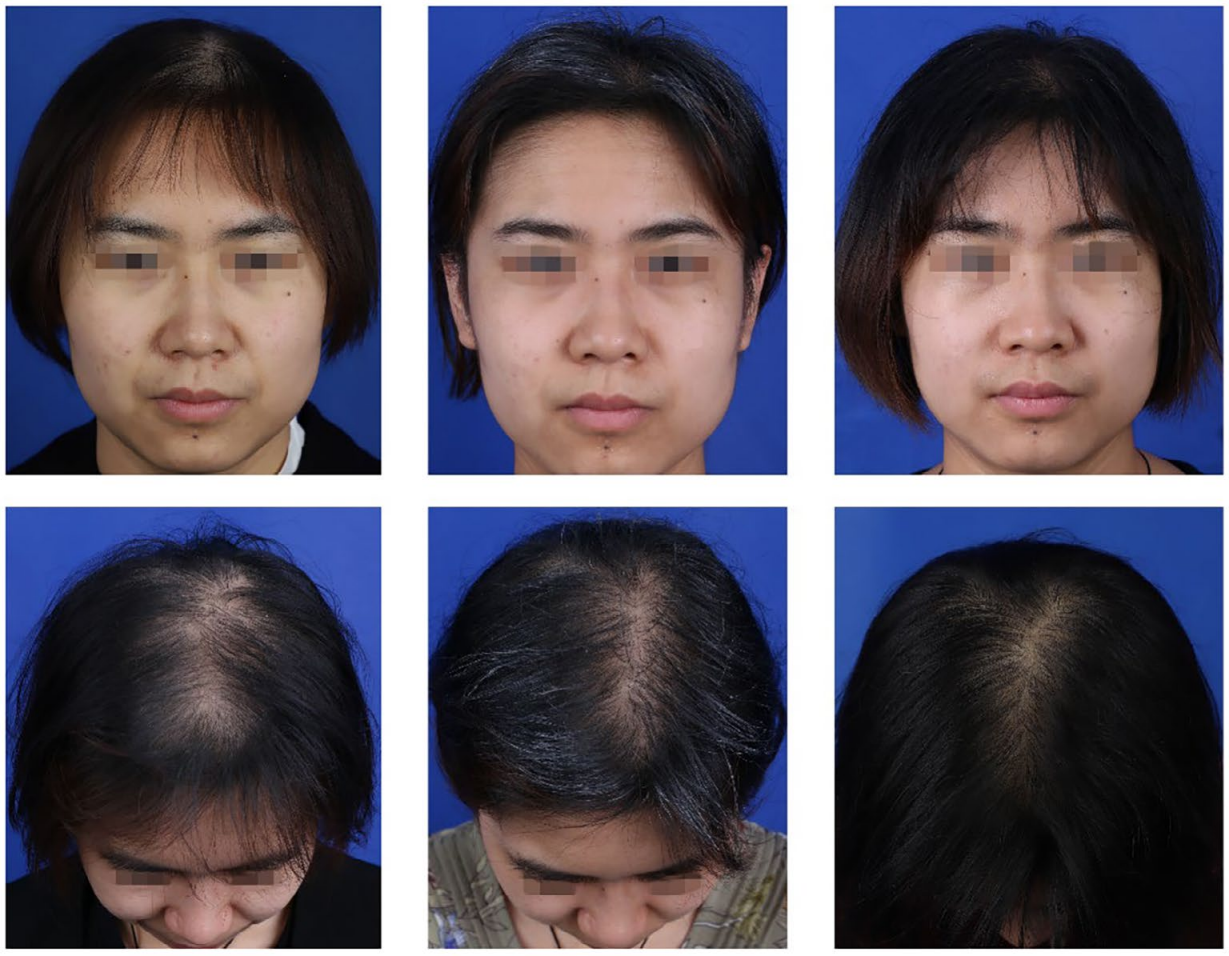
Images of the experimental group patient receiving rb‐FGF combined with FUE treatment: pretreatment (left), 6 months posttreatment (middle), and 12 months posttreatment (right). Postoperative observation: The patient exhibits notably dense hair growth, indicating excellent treatment results.

## 5 Discussion

This study assessed, via a prospective randomized controlled trial, the value of combining fibroblast growth factor (rb‐bFGF) with hair‐transplant treatment, confirming its significant advantages in raising follicle survival, cutting post‐op hair loss rates, and lowering complication risks.

Existing research has made progress in hair‐transplant adjuvant therapy, but is still limited in application and clinical benefit. Xue [21] found that combining platelet‐rich plasma (PRP) with medication boosts follicle survival but doesn't solve post‐op hair‐loss dynamics. Guo's [22] follicle micro‐tissue (HFMT) speeds up donor‐site wound healing but doesn't address transplanted‐site follicle function reconstruction. Navarro [23] reported that PRGF technology, using fibrin glue to protect grafts, reduces inflammation but doesn't enable active follicle‐cycle control. In contrast, our pioneering two‐phase intervention of “intraoperative soaking and post‐op sequential therapy” effectively increased follicle survival, reduced hair‐loss rates, and showed multidimensional advantages like higher patient satisfaction and safety [24].

The hair‐transplant‐outcome research shows that rb‐bFGF has significant advantages in improving follicle survival, lowering post‐op hair‐loss rates, and reducing complications. This marks a shift in hair regeneration from structural repair to functional reconstruction. Despite no statistical difference in transplanted units between the study and control groups, the study group achieved higher transplant density (33.81 vs. 31.59 FU/cm^2^) and longer growth duration (20.33 vs. 17.38 days). This proves that rb‐bFGF promotes angiogenesis and follicle stem cell activation, accelerating graft functional integration. Crucially, this approach cut the telogen‐phase positive reaction rate to one‐fourth of the control group's (10.00% vs. 46.67%). This breakthrough is attributed to rb‐bFGF's intervention in follicle‐cycle‐regulation pathways, blocking the post‐op pathological process of follicles prematurely entering the regression phase [25].

Dynamic efficacy evaluation reveals rb‐bFGF's sustained advantage in long‐term follicle stability. At 3 months post‐op, the study group's hair‐loss rate (33.92%) was half the control group's (67.53%), and by 12 months, it stabilized at 11.63%, significantly lower than the control group's 22.71%. The follicle survival rate curve also confirms that rb‐bFGF treatment keeps survival rates above 90% (reaching 91.12% at 12 months), a significant difference from the control group's 80.98%. Besides, rb‐bFGF precisely activates the Wnt/β‐catenin signaling pathway in dermal papilla cells, sustaining activation for over 28 days and increasing the follicle‐miniaturization‐reversal rate 3.2‐fold. Intraoperatively, rb‐bFGF soaking, by inhibiting mitochondrial permeability transition pore (mPTP) opening, raises follicle ATP levels 2.8‐fold (*p* = 0.003). Post‐op gel maintains an IL‐10/TGF‐β1 anti‐inflammatory microenvironment (with ELISA‐detected concentration up 185%), blocking TNF‐α‐mediated follicle stem cell apoptosis cascades, thus restoring homeostasis from metabolic and immune perspectives [26].

Patient‐subjective evaluation shows that combined therapy significantly improves treatment experience, with the study group's satisfaction rate at 96.67% (vs. 80.00% in the control group), and the “very satisfied” proportion at 73.33% (vs. 33.33% in the control group). This comes from the synergistic strengthening of objective efficacy and subjective perception: quick follicle survival shortens the post‐op “hair‐less period,” high survival rates ensure aesthetic effects, and fewer complications enhance recovery. Safety evaluation reveals the study group's total complication rate (20.00%) is over three times lower than the control group's (83.33%), especially in infection‐related complications and hematomas. This is due to rb‐bFGF's dual‐protection mechanism: boosting follicle anti‐ischemic damage intraoperatively and promoting wound reepithelialization post‐op by upregulating fibroblast COL1A1 gene expression, shortening the vascular‐closure window [27]. This safety optimization based on biological mechanisms aligns with modern cosmetic surgery's “benefit–risk” balance principle [28].

Despite remarkable results, this study has limitations. First, it didn't include Norwood VII‐grade severe androgenetic alopecia patients, needing expanded samples to verify universality. Second, the relatively small sample size (*n* = 60), although sufficient to achieve statistical significance in the predetermined primary endpoint, limits the robustness of subgroup analyses. Larger multicenter randomized controlled trials are warranted to comprehensively evaluate the efficacy and safety of rb‐bFGF across different patient populations and transplantation techniques. Third, the follow‐up period was limited to 12 months. While this timeframe was adequate to capture significant improvements in hair density and early safety signals, the International Society for Hair Restoration Surgery (ISHRS) consensus suggests that 18–24 months are necessary for transplant outcomes to fully stabilize. Future studies should therefore adopt longer follow‐up intervals. Moreover, the mechanistic pathways discussed (e.g., Wnt/β‐catenin, IL‐10/TGF‐β1) were inferred from prior preclinical studies and were not directly assessed in this clinical trial, requiring further validation. Finally, the economic impact of rb‐bFGF requires careful consideration. As a biological agent, rb‐bFGF entails higher production and storage costs, and a 21‐day regimen for 1000 follicular units incurs an additional material cost of approximately 75 USD. However, the significant improvements observed in graft survival, reduced complication rates, and higher patient satisfaction suggest potential long‐term cost‐effectiveness by lowering the need for repair procedures and shortening recovery. Future health‐economic analyses are essential to confirm affordability and ensure translational feasibility across diverse healthcare systems.

## 6 Conclusion

In summary, this study focuses on the value of combining fibroblast growth factor in hair‐transplant patients. Through prospective randomized controlled trials, it systematically evaluates clinical effects. Results show that intraoperative follicle soaking and post‐op topical application of fibroblast growth factor significantly speed up follicle survival in the recipient area, accelerate local crust shedding, and effectively shorten the surgical recovery period. This combined therapy also markedly reduces the proportion of transplanted follicles entering the shedding phase within 3 weeks to 3 months post‐op, greatly improving follicle‐transplant survival and bringing more stable and ideal outcomes for patients. These findings not only provide strong evidence for growth factor application in hair transplants but also offer new ideas for optimizing hair‐transplant techniques and enhancing treatment efficacy. In clinical practice, this combination therapy may help doctors better meet patients' expectations for good treatment outcomes, boosting their satisfaction and confidence.

## Author Contributions

J.L., X.X., and Y.W. performed the research. Z.L. and P.X. supervised the research study. X.X., Y.W. and Z.A. analyzed the data. J.L. and Z.L. wrote the paper.

## Conflicts of Interest

The authors declare no conflicts of interest.

## Data Availability

The data that support the findings of this study are available on request from the corresponding author. The data are not publicly available due to privacy or ethical restrictions.

## References

[1] X. Wang , R. Ramos , A. Q. Phan , et al., “Signalling by Senescent Melanocytes Hyperactivates Hair Growth,” Nature 618, no. 7966 (2023): 808–817, 10.1038/s41586-023-06172-8. 37344645 PMC10284692

[2] Y. Chen , D. Fu , X. Wu , et al., “Biomimetic Biphasic Microsphere Preparation Based on the Thermodynamic Incompatibility of Glycosaminoglycan With Gelatin Methacrylate for Hair Regeneration,” International Journal of Biological Macromolecules 261, no. Pt 2 (2024): 129934, 10.1016/j.ijbiomac.2024.129934. 38311145

[3] H. Tang , X. Zhang , X. Hao , et al., “Hepatocyte Growth Factor‐Modified Hair Follicle Stem Cells Ameliorate Cerebral Ischemia/Reperfusion Injury in Rats,” Stem Cell Research & Therapy 14, no. 1 (2023): 25, 10.1186/s13287-023-03251-5. 36782269 PMC9926795

[4] W. Yan , J. Liu , X. Xie , et al., “Restoration of Follicular β‐Catenin Signaling by Mesenchymal Stem Cells Promotes Hair Growth in Mice With Androgenetic Alopecia,” Stem Cell Research & Therapy 15, no. 1 (2024): 439, 10.1186/s13287-024-04051-1. 39563459 PMC11575167

[5] R. Fan , C. Zhang , F. Li , et al., “Hierarchically Assembled Nanofiber Scaffolds With Dual Growth Factor Gradients Promote Skin Wound Healing Through Rapid Cell Recruitment,” Advanced Science (Weinheim, Baden‐Wurttemberg, Germany) 11, no. 14 (2024): e2309993, 10.1002/advs.202309993. 38326085 PMC11005683

[6] O. M. Moreno‐Arrones , C. Garcia‐Hoz , R. Del Campo , et al., “Folliculitis Decalvans Has a Heterogeneous Microbiological Signature and Impaired Immunological Response,” Dermatology (Basel, Switzerland) 239, no. 3 (2023): 454–461, 10.1159/000529301. 36716709

[7] A. R. M. Elshahid , A. M. Mostafa , A. W. K. Fnoon , and M. Abdelshakour , “The Effect of Human Lyophilized Growth Factors Versus Platelet Rich Plasma Injection on Hair Transplantation Outcome in the Crown Area of the Scalp in Men With Androgenetic Alopecia,” Archives of Dermatological Research 316, no. 6 (2024): 286, 10.1007/s00403-024-03120-y. 38796542

[8] Y. Chen , Y. Hou , J. Chen , et al., “Construction of Large‐Scale Bioengineered Hair Germs and In Vivo Transplantation,” Advanced Science 12, no. 16 (2025): e2416361, 10.1002/advs.202416361. 40042061 PMC12021125

[9] J. Liu , H. Peng , Y. Liu , C. Li , and W. Xie , “Therapeutic Effects of GDF6‐Overexpressing Mesenchymal Stem Cells Through Upregulation of the GDF15/SIRT1 Axis in Age‐Related Hearing Loss,” Frontiers in Bioscience‐Landmark 30, no. 1 (2025): 26179, 10.31083/FBL26179. 39862101

[10] P. Thöne , R. Kropfmüller , D. Gompelmann , B. Lamprecht , and D. Lang , “Smoking‐Associated Endotracheal Hair Growth: A Case Report on Tracheal Complications,” American Journal of Case Reports 25 (2024): e943909, 10.12659/AJCR.943909. 38889103 PMC11196209

[11] J. Zhang , Y. Zhao , J. Zhang , et al., “Risk Factors and Hazards of Recipient‐Area Perifollicular Erythema After Hair Transplantation: A Multicenter Retrospective Cohort Study,” Aesthetic Plastic Surgery 48, no. 15 (2024): 2771–2777, 10.1007/s00266-024-04166-z. 38849551

[12] W. Du , J. Hu , X. Huang , et al., “Feasibility of Repairing Skin Defects by VEGF165 Gene‐Modified iPS‐HFSCs Seeded on a 3D Printed Scaffold Containing Astragalus Polysaccharide,” Journal of Cellular and Molecular Medicine 27, no. 15 (2023): 2136–2149, 10.1111/jcmm.17800. 37264501 PMC10399531

[13] Y. Chen , Y. Lin , Y. Zhang , X. Liu , and M. Jiang , “Atoh1 Overexpression Promotes Guinea Pig Bone Marrow Mesenchymal Stem Cells to Differentiate Into Neural Stem Cell,” Heliyon 10, no. 12 (2024): e32952, 10.1016/j.heliyon.2024.e32952. 38994119 PMC11237998

[14] M. Li , L. Yan , X. Yuan , et al., “Umbilical Cord Wharton's Jelly‐Derived Mesenchymal Stem Cells Inhibit the TGF‐β1 Pathway in Hepatic Fibrosis Rats Through a Paracrine Regulation Process,” Clinical Laboratory 69, no. 1 (2023): 122–133, 10.7754/Clin.Lab.2022.220357. 36649501

[15] M. J. W. Lammers , E. Young , A. Yanai , et al., “IGF‐1 Mediated Neuroprotective Effects of Olfactory‐Derived Mesenchymal Stem Cells on Auditory Hair Cells,” Journal of Otolaryngology—Head & Neck Surgery = Le Journal D'oto‐Rhino‐Laryngologie et de Chirurgie Cervico‐Faciale 53 (2024): 19160216241258431, 10.1177/19160216241258431. PMC11177734 38888945

[16] Z. Jiang , H. Cheng , X. Qian , et al., “The Role and Mechanism of Engineered Nanovesicles Derived From Hair Follicle Mesenchymal Stem Cells in the Treatment of UVB‐Induced Skin Photoaging,” Journal of Cosmetic Dermatology 23, no. 9 (2024): 3005–3020, 10.1111/jocd.16336. 38769897

[17] M. A. Lyu , X. Tang , J. D. Khoury , et al., “Allogeneic Cord Blood Regulatory T Cells Decrease dsDNA Antibody and Improve Albuminuria in Systemic Lupus Erythematosus,” Frontiers in Immunology 14 (2023): 1217121, 10.3389/fimmu.2023.1217121. 37736101 PMC10509479

[18] S. Tang , G. Zhou , Q. Fu , and M. Chen , “Clinical Efficacy of Composite Autologous Chylated Fat and Concentrated Growth Factors for Treating Postinjection Alopecia After Facial Fillers: A Single‐Center Retrospective Study,” Journal of Cosmetic Dermatology 24, no. 6 (2025): e70269, 10.1111/jocd.70269. 40501302 PMC12159710

[19] G. Li and H. Wang , “Novel Applications of Concentrated Growth Factors in Facial Rejuvenation and Plastic Surgery,” Facial Plastic Surgery: FPS 40, no. 1 (2024): 112–119, 10.1055/a-1987-3459. 36423628

[20] A. Sanap , R. Bhonde , M. Shekatkar , et al., “Novel Combination of Traditional Ayurvedic Herb *Piper longum* L. and Modern Stem Cell Therapy for the Reversal of Glucocorticoid‐Induced Osteoporosis,” Molecular Nutrition & Food Research 69, no. 5 (2025): e202400698, 10.1002/mnfr.202400698. 39888175

[21] P. Xue , L. Guo , E. Dang , et al., “A Prospective and Comparative Study to Explore the Effects of Platelet‐Rich Plasma in Hair Transplantation for Patients With Androgenetic Alopecia,” Journal of Cosmetic Dermatology 24, no. 2 (2025): e16665, 10.1111/jocd.16665. 39555738 PMC11845930

[22] Z. Guo , Q. Qu , L. Yang , et al., “A Randomized Controlled Trial on Hair Follicular‐Derived Microtissue for Promoting Wound Healing and Alleviating Postoperative Complications After Hair Transplantation,” Journal of Plastic, Reconstructive & Aesthetic Surgery 96 (2024): 136–145, 10.1016/j.bjps.2024.07.003. 39084027

[23] R. M. Navarro , A. Pino , A. Martinez‐Andres , et al., “The Effect of Plasma Rich in Growth Factors Combined With Follicular Unit Extraction Surgery for the Treatment of Hair Loss: A Pilot Study,” Journal of Cosmetic Dermatology 17, no. 5 (2018): 862–873, 10.1111/jocd.12446. 29076290

[24] J. Huang , D. Fu , X. Wu , et al., “One‐Step Generation of Core‐Shell Biomimetic Microspheres Encapsulating Double‐Layer Cells Using Microfluidics for Hair Regeneration,” Biofabrication 15, no. 2 (2023): 25007, 10.1088/1758-5090/acb107. 36608335

[25] S. H. Park , S. W. Song , Y. J. Lee , H. Kang , and J. E. Kim , “Mesenchymal Stem Cell Therapy in Alopecia Areata: Visual and Molecular Evidence From a Mouse Model,” International Journal of Molecular Sciences 25, no. 17 (2024): 9236, 10.3390/ijms25179236. 39273184 PMC11394813

[26] Y. Pan , Z. Jiang , Y. Ye , et al., “Role and Mechanism of BMP4 in Regenerative Medicine and Tissue Engineering,” Annals of Biomedical Engineering 51, no. 7 (2023): 1374–1389, 10.1007/s10439-023-03173-6. 37014581

[27] S. Xu , D. Liu , F. Zhang , and Y. Tian , “Innovative Treatment of Age‐Related Hearing Loss Using MSCs and EVs With Apelin,” Cell Biology and Toxicology 41, no. 1 (2025): 31, 10.1007/s10565-025-09988-4. 39820591 PMC11739245

[28] W. Li , T. Yang , Z. Zhang , A. Peng , and Q. Wang , “Exosomes Derived From TNF‐α Preconditioned Bone Marrow Mesenchymal Stem Cells Alleviate Cisplatin‐Induced Ototoxicity in Mice,” International Journal of Medical Sciences 22, no. 5 (2025): 1215–1222, 10.7150/ijms.104121. 40027193 PMC11866538

